# Antiviral mechanisms of guanylate-binding protein 5: versatile inhibition of multiple viral glycoproteins

**DOI:** 10.1128/mbio.02374-24

**Published:** 2024-10-15

**Authors:** Daniel Sauter, Frank Kirchhoff

**Affiliations:** 1 Institute for Medical Virology and Epidemiology of Viral Diseases, University Hospital Tübingen, Tübingen, Germany; 2 Institute of Molecular Virology, Ulm University Medical Center, Ulm, Germany; Columbia University Medical Center, New York, New York, USA

**Keywords:** guanylate-binding protein 5, innate immunity, viral envelope proteins

## Abstract

Guanylate-binding proteins (GBPs) are interferon-inducible cellular factors known to inhibit a wide variety of pathogens. Humans encode seven GBPs that have functionally diversified to provide broad protection against a variety of bacteria, protozoa, and viruses. Here, we discuss recent data on the mechanisms underlying the broad antiviral activity of GBP5 (H. Veler, C. M. Lun, A. A. Waheed, and E. O. Freed, mBio e02086-24, 2024, https://doi.org/10.1128/mbio.02086-24) and place them in the context of previous studies on the ability of this antiviral factor to impair the function of numerous viral envelope (Env) glycoproteins. We focus on the effects of GBP5 on the glycosylation, proteolytic processing, and anterograde transport of Env and discuss mechanistic interdependencies of these maturation steps. Understanding the induction and action of broadly acting immune factors, such as GBP5, may help develop effective immune-based strategies against numerous pathogens.

## COMMENTARY

**FIG 1:**
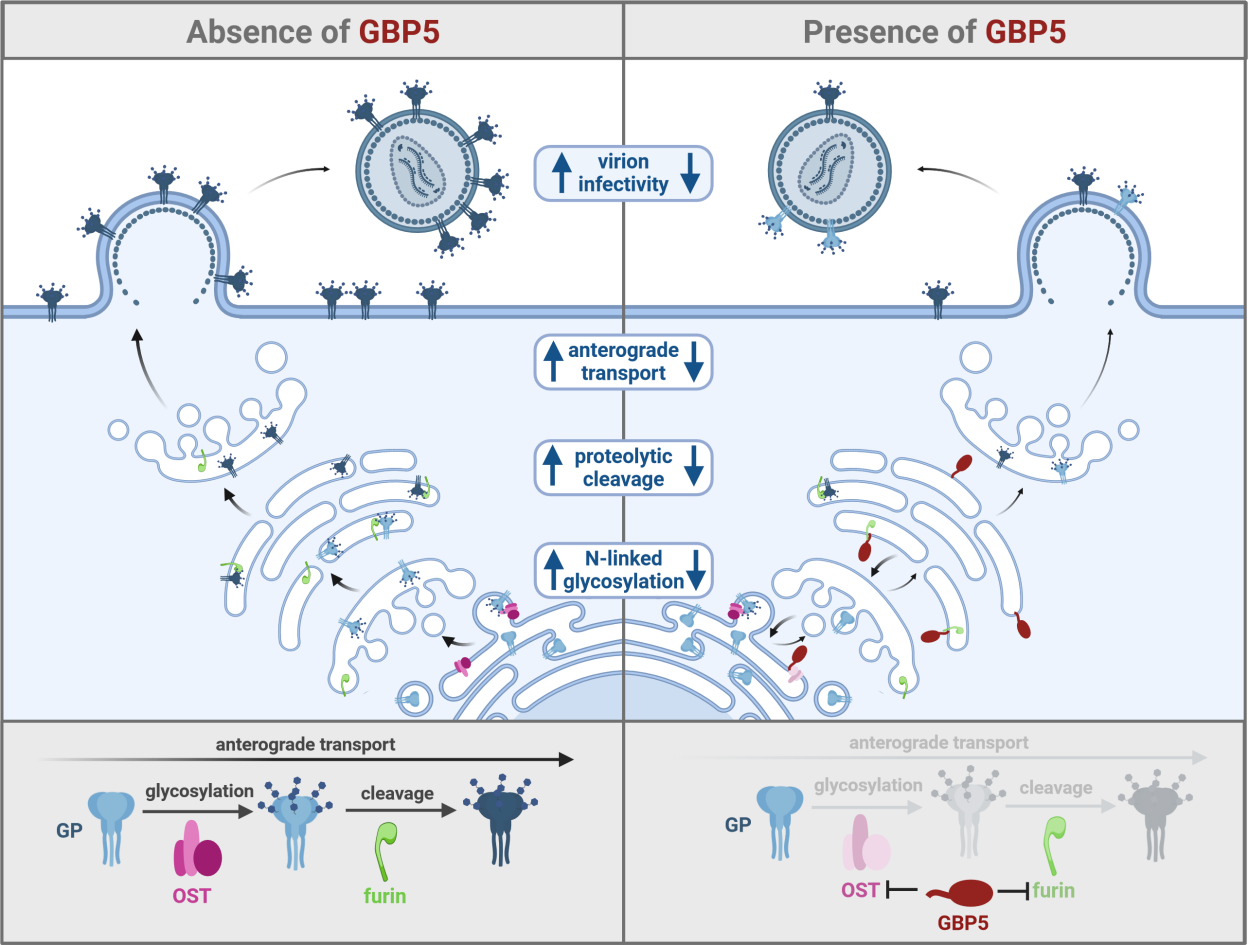
GBP5-mediated restriction of viral glycoprotein function. GBP5 reduces virion infectivity of various viral pathogens by inhibiting the (i) anterograde transport, (ii) N-linked glycosylation, and/or (iii) proteolytic processing of viral glycoproteins (GP). The host protease furin (green) and the oligosaccharyltransferase complex (OST, pink) have been described as targets of GBP5 (red), resulting in impaired proteolytic processing and glycosylation of viral glycoproteins (blue), respectively. The figure was generated using BioRender.

To achieve broad protection against the myriad of highly diverse pathogens, the innate immune system has evolved numerous sophisticated defense mechanisms. These include antiviral restriction factors, which often target conserved viral components or commonly exploited cellular dependency factors. In addition, host genes encoding innate defense factors have often multiplied and functionally diversified to keep up in the arms race with harmful pathogens. Guanylate-binding proteins (GBPs) are a family of interferon (IFN)-inducible guanosine triphosphatases (GTPases) that have long been known to sense and inhibit various intracellular bacterial and protozoan pathogens, including *Toxoplasma gondii*, *Chlamydia trachomatis*, *Legionella spec*., and *Mycobacterium tuberculosis* (1). More recently, some members of the GBP family have also been shown to inhibit viral pathogens. Initially, human GBP5 was reported to share features of previously known restriction factors (2) and restrict HIV-1 by interfering with the anterograde transport, proteolytic processing, and virion incorporation of the viral envelope (Env) glycoprotein (3) (Fig. 1). Subsequent studies suggested that human GBP5 and its paralogue GBP2 inhibit furin, a cellular protease that is critical for the proteolytic cleavage and function of many viral Env glycoproteins (4, 5). In support of a relevant role of furin inhibition in antiviral activity, GBP2 and GBP5 restrict a variety of furin-dependent viruses, including HIV-1, measles virus, Zika virus, highly pathogenic avian influenza A viruses, human endogenous retrovirus K (HERV-K), and severe acute respiratory syndrome coronavirus 2 (SARS-CoV-2) (6–8).

Veler et al. confirmed the ability of GBP5 to impair the infectivity of furin-dependent viruses (9). However, in contrast to previous studies (4, 8), they found that GBP5 also interfered with the function of the Env glycoproteins from vesicular stomatitis virus (VSV) and SARS-CoV-1, which do not require furin to mediate infection. In agreement with the results of a recent preprint (10), their results suggest that GBP5 impairs viral infectivity by interfering with the trafficking and N-linked glycosylation of viral Env glycoproteins rather than by directly inhibiting furin activity. Notably, the effects of GBP5 on HIV-1 Env trafficking, glycosylation, cell surface expression, and virion incorporation of viral Env glycoproteins reported by Veler and colleagues all agree with previous data (3–5). In fact, comprehensive structural and functional analyses identified two mutations (L307A and P308A) that had little effect on GBP5’s ability to inhibit furin and proteolytic HIV-1 Env processing, but impaired its ability to reduce HIV-1 infectivity, suggesting restriction by genetically separable furin-dependent and -independent mechanisms (5).

While all these studies agree that GBP5 affects N-linked glycosylation, trafficking, and virion incorporation of viral glycoproteins, there is controversy about the role of furin in the restriction of viruses that are dependent on this cellular protease (Table 1). Veler et al. did not observe an effect of GBP5 on furin-mediated processing of HIV-1 Env glycoproteins (9). In contrast, we detected a significant effect on the processing of the HIV-1 gp160 Env precursor to functional gp120 even when all glycosylated forms of gp120 or PNGase F-digested gp120 were considered (3). Similarly, Veler et al. reported no effect of GBP5 on SARS-CoV-2 Spike cleavage, while an earlier study by the Jolly lab did (8). Since furin itself is also N-glycosylated, one might speculate that furin inhibition is merely a side effect of altered glycosylation in the presence of GBP5. However, this is unlikely since furin co-immunoprecipitates with GBP5 (4) and both functions are genetically separable (5). In addition, a specific fluorescence-based reporter assay showed that GBP5 inhibits the proteolytic activity of human furin (4, 5). Still, it is important to note that N-linked glycosylation, proteolytic protein cleavage, and anterograde transport can mutually affect each other. For example, Wang and colleagues suggest that GBP5 may not directly affect furin activity but impact the proteolytic cleavage of viral glycoproteins by affecting their trafficking to the Golgi compartment (10). Finally, other members of the proprotein convertase family that are not inhibited by GBP5 might compensate for the lack of furin activity under some experimental conditions.

**TABLE 1:** Reported effects of GBP5 on furin and/or viral glycoprotein cleavage^*a*^

Readout	Outcome	GBP5 modulation	Cell type	Study
Ratio of cleaved to uncleaved viral glycoprotein (western blot)	Reduced (HIV-1 Env in virions)	Overexpression	HEK293T	Krapp et al. (3)
	Reduced (MLV Env in cell lysates)	Overexpression	HEK293T	Krapp et al. (3)
	Reduced (HIV-1 Env in virions)	Overexpression	HEK293T	Braun et al. (4)
	Reduced (HIV-1 Env and IAV HA in cell lysates)	Overexpression	HEK293T	Braun et al. (4)
	Reduced (HIV-1 Env in virions)	Overexpression	HLAC	Braun et al. (4)
	Reduced (HERV-K Env in cell lysates)	Overexpression	HEK293T	Srinivasachar et al. (7)
	Reduced (HIV-1 Env in virions)	Overexpression	HEK293T	Cui et al. (5)
	Reduced (SARS-CoV-2 S in pseudovirions)	Overexpression	HEK293T	Mesner et al. (8)
	Reduced (SARS-CoV-2 S in cell lysates)	Overexpression	HEK293T	Mesner et al. (8)
	Reduced (SARS-CoV-2 S in cell lysates)	Overexpression	HEK293T	Wang et al. (10)
	Reduced (SARS-CoV-2 S in virions)	Overexpression	HEK293T	Wang et al. (10)
	Reduced (HIV-1 Env, HIV-1 Env, MLV Env, and SARS-CoV-2 S in pseudovirions)	Overexpression	HEK293T	Veler et al. (9)
	Unchanged (HIV-1 Env, MLV Env, and SARS-CoV-2 S in cell lysates)	Overexpression	HEK293T	Veler et al. (9)
	Unchanged (SARS-CoV-2 S in VLPs)	Overexpression	HEK293T	Want et al. (10)
	Increased (HIV-1 Env in cell lysates)	Knockdown	MDM	Krapp et al. (3)
	Increased (SARS-CoV-2 S in virions)	Knockdown	Calu-3	Mesner et al. (8)
Interaction of GBP5 and furin (Co-IP)	Precipitation of furin with GBP5	Overexpression	HEK293T	Braun et al. (4)
	Precipitation of GBP5 with furin	None	MDM	Braun et al. (4)
Protease activity (AMC reporter assay)	Reduced cleavage of a furin reporter substrate by cell supernatants and lysates	Overexpression	HEK293T	Braun et al. (4)
	Reduced proteolytic activity of furin isolated from cell lysates	Overexpression	HEK293T	Cui et al. (5)
	Reduced cleavage of a furin reporter substrate by culture supernatants	Overexpression	HEK293T	Schelle et al. (11)
	Increased cleavage of a furin reporter substrate by cell lysates	Knockout (+IFNγ)	THP1	Braun et al. (4)

^*a*^ AMC, 7-amino-4-methylcoumarin; Co-IP, co-immunoprecipitation; Env, envelope protein; HA, hemagglutinin; HERV-K, human endogenous retrovirus K; HIV-1, human immunodeficiency virus type I; HEK293T cells, human embryonic kidney 293T cells; HLAC, human lymphoid aggregate culture; IAV, influenza A virus; IFNγ, interferon γ; MDM, monocyte-derived macrophages; MLV, murine leukemia virus; S, Spike protein; SARS-CoV-2, severe acute respiratory syndrome coronavirus 2; VLP, virus-like particle.

In line with a significant selection pressure exerted by GBP5, some viruses have evolved evasion (3, 8) or counteraction (12) strategies. For example, the D614G mutation in SARS-CoV-2 Spike rendered the Alpha and Delta variants resistant to GBP5-mediated restriction, most likely as a result of generally increased virion infectivity (8). Similarly, Veler et al. show that high levels of HIV-1 Env and SARS-CoV-2 Spike expression overcome GBP5 restriction. This agrees with the previous finding that some macrophage-tropic HIV-1 strains evade GBP5 restriction by an unusual trade-off mechanism, that is, elimination of the *vpu* ATG initiation codon, to increase the levels of Env expressed from the same bicistronic RNA (3). The fact that antiviral effects depend on the levels of GBP5 and Env expression may also explain some seeming discrepancies. A limitation of the study by Veler et al., as well as earlier publications, is that many results were obtained in transfected HEK293T cells using lentivirus-based pseudo-particles (Table 1). While Veler and colleagues made an effort to mimic the GBP5 expression levels found in primary T cells, the experimental conditions may still not fully recapitulate the spectrum of GBP5 activities in primary target in the presence of different levels of IFNs and other cytokines. Notably, it has previously been shown that endogenous GBP5 protein levels inversely correlate with infectious HIV-1 production in primary human macrophages, strongly supporting a relevant role *in vivo* (3). Conversely, *GBP5* knockdown in macrophages markedly increased gp120 levels in HIV-1 progeny virions and was associated with enhanced proteolytic processing of the gp160 Env precursor (3). To further elucidate the antiviral mechanism(s) of GBP5 and its role in antiviral immunity, it will be critical to determine the effect of GBP5 on genuine viruses in their respective primary target cells and in appropriate animal models. GBP5 also affects the trafficking, glycosylation, and proteolytic processing of cellular proteins (6). Thus, studies approximating *in vivo* conditions will also help clarify whether GBP5 affects viral glycoproteins at lower expression levels than cellular factors to minimize adverse effects.

The new study by Veler and colleagues adds to the evidence that restriction factors targeting viral glycoproteins act by various mechanisms to achieve broad and effective antiviral protection. For example, MARCH8 was initially reported to inhibit viral pathogens by reducing virion incorporation of HIV-1 and VSV glycoproteins (13). However, recent studies suggest that MARCH8 also inhibits the proteolytic activity of furin (14, 15). Thus, comparative analyses of antiviral factors targeting viral glycoproteins (16, 17), including those that inhibit proteolytic glycoprotein activation, are clearly warranted. It will be of interest to further elucidate how these factors acting by similar or overlapping mechanisms may synergize to reduce viral infectivity and replication. A better understanding of the regulation, modes of action, and interaction of broadly active innate antiviral factors may help develop safe and effective therapeutic approaches.
